# Supplementary figures and images for: Digital Pathology Applications for PD-L1 Scoring in Head and Neck Squamous Cell Carcinoma: A Challenging Series
Source: J Clin Med. 2024 Feb 22;13(5):1240. doi: 10.3390/jcm13051240 (PMC10932078; doi:10.3390/jcm13051240)

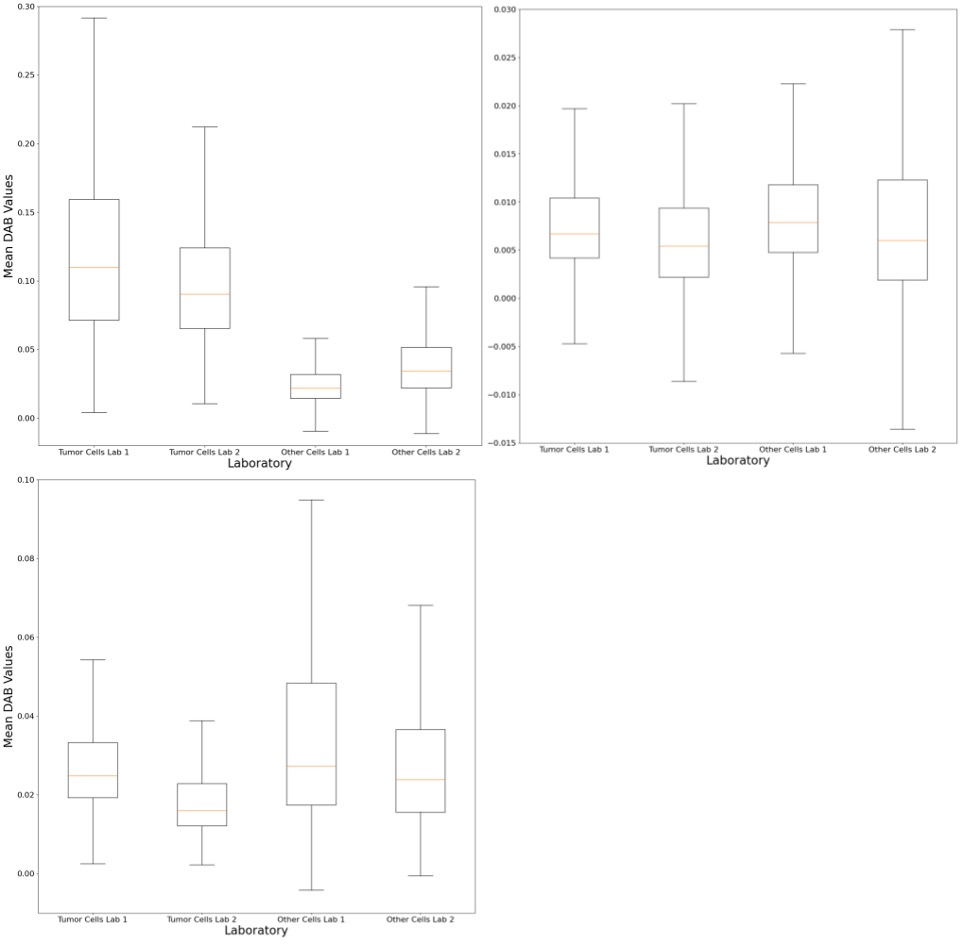

Supplement: Supplementary file 1 [file jcm-13-01240-s001.zip › Supplementary Figure S1.jpg]
